# Characterization of a KANADI-like transcription factor that suppresses pear anthocyanin biosynthesis

**DOI:** 10.1093/hr/uhaf071

**Published:** 2025-03-03

**Authors:** Weilin Wei, Kui Lin-Wang, Guosong Chen, Richard V Espley, Andrew C Allan, Beibei Cao, Mengfan Qin, Shoufeng Sha, Juncai Li, Runze Wang, Jiaming Li, Jun Wu

**Affiliations:** College of Horticulture, State Key Laboratory of Crop Genetics and Germplasm Enhancement, Nanjing Agricultural University, Weigang Road No.1, Xuanwu District, Nanjing, Jiangsu 210095, China; The New Zealand Institute for Plant & Food Research Ltd, Mt Albert Research Centre, Private Bag, Auckland 92169, New Zealand; College of Horticulture, State Key Laboratory of Crop Genetics and Germplasm Enhancement, Nanjing Agricultural University, Weigang Road No.1, Xuanwu District, Nanjing, Jiangsu 210095, China; The New Zealand Institute for Plant & Food Research Ltd, Mt Albert Research Centre, Private Bag, Auckland 92169, New Zealand; The New Zealand Institute for Plant & Food Research Ltd, Mt Albert Research Centre, Private Bag, Auckland 92169, New Zealand; College of Horticulture, State Key Laboratory of Crop Genetics and Germplasm Enhancement, Nanjing Agricultural University, Weigang Road No.1, Xuanwu District, Nanjing, Jiangsu 210095, China; College of Horticulture, State Key Laboratory of Crop Genetics and Germplasm Enhancement, Nanjing Agricultural University, Weigang Road No.1, Xuanwu District, Nanjing, Jiangsu 210095, China; Division of Pear Breeding, Institute of Pomology, Liaoning Academy of Agricultural Sciences, Tiedong Road, Xiongyue, Yingkou, Liaoning 115009, China; Division of Pear Breeding, Institute of Pomology, Liaoning Academy of Agricultural Sciences, Tiedong Road, Xiongyue, Yingkou, Liaoning 115009, China; College of Horticulture, State Key Laboratory of Crop Genetics and Germplasm Enhancement, Nanjing Agricultural University, Weigang Road No.1, Xuanwu District, Nanjing, Jiangsu 210095, China; College of Horticulture, State Key Laboratory of Crop Genetics and Germplasm Enhancement, Nanjing Agricultural University, Weigang Road No.1, Xuanwu District, Nanjing, Jiangsu 210095, China; College of Horticulture, State Key Laboratory of Crop Genetics and Germplasm Enhancement, Nanjing Agricultural University, Weigang Road No.1, Xuanwu District, Nanjing, Jiangsu 210095, China

## Abstract

Anthocyanins are important specialized fruit metabolites and major pigments, whose abundance depends on co-regulation of activators and repressors, primarily transcription factors (TFs) of the MYB family. Herein, a KANADI-like TF PuKAN4 was characterized in pear. This TF could be transcriptionally up-regulated by the anthocyanin-related R2R3-MYBs PuMYB10/PuMYB114 and exhibited high expression within red-skinned pears. Interestingly, *PuKAN4* repressed anthocyanin biosynthesis in transiently overexpressed pear fruit, and stable transformation in pear calli and tobacco plants. The PuKAN4 had a conserved EAR repression domain in C-terminal*,* while the repression function of PuKAN4 could be offset by a transcription activation domain VP64. The dual luciferase analysis proved that PuMYB114/PuMYB10 up-regulated expression of *PuKAN4.* Furthermore, the PuKAN4 could physically interact with PuMYB10/PuMYB114 and did not affect the combination of MYB10/MYB114-bHLH3, as demonstrated by Y2H, pull-down and firefly luciferase complementation. Thus, the PuKAN4 should play the role of active repressor, the formation of PuKAN4-PuMYB10/PuMYB114-PubHLH3 complex inhibited pear anthocyanin biosynthesis. Our findings unveiled an activator-and-repressor feedback loop between PuMYB114/PuMYB10 and PuKAN4, which possibly balance biosynthesis activity and prevents over-accumulation of phenylpropanoids.

## Introduction

Anthocyanins, a subgroup of flavonoids, are extensively dispersed throughout plant organs, namely the flowers, leaves, roots, seeds, and fruits [1]. Anthocyanins play diverse roles during various plant physiological processes, including resistance to biotic and abiotic stresses, attracting pollinator, seed dispersal, and protection against UV light [2–4]. Dietary anthocyanins also have benefits to human health [5, 6].

Previous research has characterized multiple enzyme-encoding genes implicated with the anthocyanin biosynthetic process [7], including early (EBGs) and late biosynthesis genes (LBGs) [8]. Among them, the characterized EBGs were *4 coumarate CoA ligase*, *chalcone isomerase*, *cinnamic acid 4-hydroxylase*, *chalcone synthase*, *flavanone 3′5′-hydroxylase* (*F3′5′H*), *flavanone 3′-hydroxylase* (*F3′H*), *flavanone 3-hydroxylase*, and *phenylalanine ammonia lyase*, which can be activated by the different R2R3-MYB factors, such as *MYB11*, *MYB12* and *MYB111* [9]. LBGs identified were *anthocyanidin synthase/leucoanthocyanidin dioxygenase*, *dihydroflavonol reductase* (*DFR*), and *UDP-flavonoid glucosyl transferase* (*UFGT*), among others, whose expression activation depends on the MBW complex [10].

The MBW complex is the critical activation system for anthocyanin biosynthesis in many species, including *Malus domestica* (apple) [11], *Pyrus* (pear) [12], *Prunus persica* (peach) [13], *Rosa rugosa* (beach rose) [14], *Fragaria* × *ananassa* (strawberry) [15], bayberry [16], and mangosteen [17]. R2R3-MYB is the most crucial MBW complex constituent. *PAP1/MYB75* overexpression in *Arabidopsis thaliana* has been shown to cause an overabundance of anthocyanins [18]. In *Petunia* × *hybrida*, *AN2* had been verified to exert regulatory effects on the accumulation of anthocyanin [19]. Investigations on many fruits have revealed the implication of R2R3-MYB TFs with the biosynthesis of anthocyanin, including apple *MYB1* [20] and *MYB10* [11], strawberry *MYB10* [21], and citrus *Ruby1* [22]. Notably, an investigation on pear showcased that *MYB10* and *MYB114* can promote anthocyanin structural genes expression through interacting with a co-regulatory protein bHLH3 [12, 23].

Apart from the aforementioned proteins activating the biosynthesis of anthocyanin in plants, a series of proteins repressing this process have also been identified [24]. MYB transcription factors, specifically R3-MYBs and R2R3-MYBs, are frequently implicated as repressors. R2R3-MYB repressors usually fall into the fourth subgroup of MYB TFs [25]. Notable examples involve *CsMYB3, FaMYB1*, *PpMYB18*, *PtrMYB182*, and *PhMYB27,* respectively, in citrus, strawberry, peach, poplar, and petunia [22, 26–29]. The R2R3-MYB repressors, which contain the bHLH-interacting motif of [D/E]LX_2_[R/K]X_3_LX_6_LX_3_R, can bind to bHLH in a competitive manner against the MBW complex, thereby repressing the biosynthesis of anthocyanin via its repression domains [22, 28, 30, 31]. The motif that interacts with bHLH exhibits high conservativeness among R3-MYB repressors, but these proteins lack motifs with repressing effects. These include *AtCPC* and *AtTRY* [32, 33], *Mimulus RTO* and *ROI1* [34, 35], tomato *SlMYBATV* [36], *Freesia hybrida FhMYBx* [30] and *Phalaenopsis PhMYBx1* [37]. These repressors occupy the binding sites in the target gene promoter or form a interaction with bHLH in a mutual exclusive manner with MYB activators.

Besides MYB repressors, there are many other mechanisms for repressing anthocyanin production. For example, apple MdHB1, an HD-ZIP type of TF, could recruit the MBW complex to the cytoplasm, thereby indirectly repressing the expression of genes regulating the biosynthesis of anthocyanin [38]. *AtSPL9* has been demonstrated to directly inhibit Arabidopsis anthocyanin biosynthesis in via destabilizing the MBW complex [39]. Apart from transcription factors, certain microRNAs, including miR828 and miR858 [40, 41], also diminish the accumulative abundance of anthocyanin through specifically degrading complex gene transcripts with regulatory effects.

**Figure 1:**
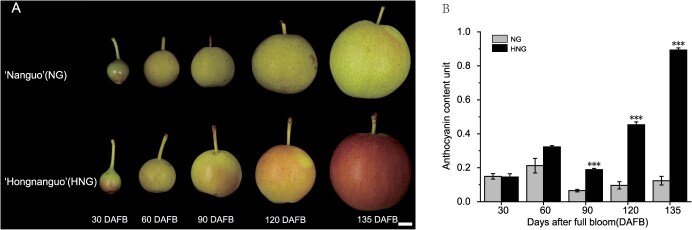
Images displaying fruits from the pear cultivars ‘Nanguo’ (NG) and ‘Hongnanguo’ (HNG) and anthocyanin content in NG and HNG. A: NG and HNG fruit phenotypes at five developmental stages, namely 30, 60, 90, 120, and 135 days after full bloom (DAFB). Scale = 1 cm. B: NG and HNG anthocyanin abundance during developmental stages. Error bars stand for the mean ± standard error (SE) of three biologically independent replicates. A two-tailed *t*-test *P* < 0.001 is presented with ***

Pear (*Pyrus spp*.) is an extensively cultivated fruit with great commercial value [42]. Among different types of pears, those with red-colored skin are very popular among customers thanks to their gleaming and attractive color [43, 44]. However, red-skinned pear is relatively rare in Asian pears, while there are many red-skinned European pears selected and cultivated [45]. Due to the limited adaptability of European pears in Asian countries, we still need to breed high-quality red-skinned Asian pears for the market. Based on previous research, different regulation modes for the coloration of Asian and European pears were identified; for example, most of Asian red-skinned pears mainly accumulate anthocyanins late during their developmental process, unlike their European counterparts accumulating anthocyanins early during their development [46, 47]. Because of the complicated genetic background of perennial fruit trees, as well as the limitation of stable transformation for gene function research [48]. Not many mutants have been utilized like in Arabidopsis to investigate the regulation mechanisms of important traits. Fortunately, fruit trees can produce natural mutants in the long-term growth, several couples of red-skinned bud mutants have been found in pears of both European and Asian origin, for example, ‘Bartlett’ versus ‘Max Red Bartlett’, ‘Early red Doyenne du Comice’ strain versus the green-colored counterpart, and ‘Zaosu’ versus ‘Red Zaosu’ [46, 49, 50]. Those mutants provide special and valuable research materials for exploring and analyzing the formation of red pear traits.

The ‘Hongnanguo’ is a natural red bud mutation derived from ‘Nanguo’ [43], which belongs to *Pyrus ussuriensis* Maxim, a widely cultivated species primarily distributed in Northeast China, particularly in extremely cold regions. Discovered and named in 2000 [51], the ‘Hongnanguo’ has gained more popularity among consumers compared to ‘Nanguo’, owing to its appealing red appearance and exceptionally juicy flavor. Nonetheless, the mechanisms regulating ‘Hongnanguo’ coloration remain largely unexplored. In this study, through the comparison of phenotype and gene expression between ‘Nanguo’ and ‘Hongnanguo’, we identified an additional repressor of anthocyanin biosynthesis, the KANADI-like transcription factor PuKAN4, which was highly expressed in red-skinned pear. A physical interaction was noted between PuKAN4 and the PuMYB10/114-PubHLH3 complex, which resulted in impaired biosynthesis of anthocyanin. The expression of *PuKAN4* was activated by PuMYB10 and PuMYB114, indicating that PuMYB10/PuMYB114 and PuKAN4 form a feedback loop that modulates anthocyanin production. Our analyses reveal the unrecognized complexity of hierarchical anthocyanin biosynthesis control mechanisms in red-skinned pears, which will provide a theoretical basis and gene resource to improve the appearance quality of the Asian pear and potentially other plant species.

## Results

### Anthocyanin levels and phenotypes in ‘Nanguo’ and ‘Hongnanguo’

‘Hongnanguo’ (HNG) is a red-skinned pear cultivar, which is a bud mutation of ‘Nanguo’ (NG). As shown in Fig. 1, during their developmental process, the peels of HNG pears gradually turned red and anthocyanin accumulation increased, with color transition and darkest red color, respectively, observed on the 90th and 135th days after full bloom (DAFB). In contrast, NG peels showed little or no red color during different developmental stages. On 90, 120, and 135 DAFB, HNG accumulated a significantly higher abundance of anthocyanin relative to NG; this was especially apparent in the last developmental stage, in which anthocyanin levels were over eight times higher in HNG than in NG (Fig. 1B). The aforementioned data revealed that the observed color differences between HNG and NG were due to peel anthocyanin accumulation.

### Analyses of differential gene expression and *PuKAN4* sequence

**Figure 2:**
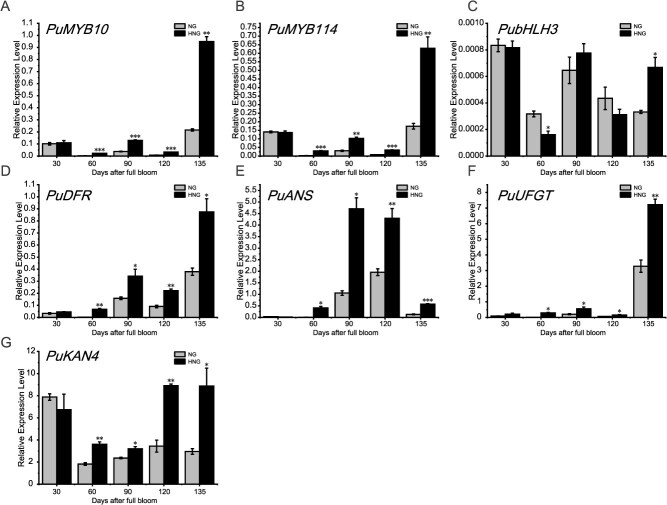
Relative *PuMYB10*, *PuMYB114*, *PubHLH3*, *PuDFR*, *PuANS, PuUFGT*, and *PuKAN4* mRNA abundance at five developmental stages. Error bars stand for the mean ± SE of three biologically independent replicates. Two-tailed *t*-test *P*-values <0.001, 0.01, and 0.05 are, respectively, marked with ***, **, and *

In a previous study, we showed that *PuMYB10*, *PuMYB114*, *PubHLH3*, *PuDFR*, *PuANS*, and *PuUFGT* are the primary regulators and key enzymes participating in pear anthocyanin biosynthesis [12]. Therefore, we subsequently measured expression levels of *PuMYB10*, *PuMYB114*, *PubHLH3*, *PuDFR*, *PuANS*, and *PuUFGT* in NG and HNG via RT-qPCR (Fig. 2A-F). The findings indicated that, compared with NG, HNG displayed superior mRNA abundance of *PuMYB10*, *PuMYB114*, *PuDFR*, and *PuANS* from 60–135 DAFB, as well as consistently higher expression of *PuUFGT* across all developmental stages. Notably, the *PubHLH3* expression level of HNG was superior relative to that in NG only on 135 DAFB.

Next, we explored genes with potential regulatory effects on pear anthocyanin biosynthesis and noted clear differences in fruit peel color between NG and HNG from the early fruit developmental stages through to fruit ripening. Using the *Pyrus bretschneideri* Rehd*.* reference genome [42], RNA-seq analysis with peel samples at 135 DAFB was conducted to identify 1555 differentially expressed genes (DEGs) through applying the *P* ≤ 0.05 and fold change <0.5 or > 2.0 cut-off standards. Among these 1555 DEGs, 794 and 761, respectively, displayed significantly elevated and reduced expression levels (Supplemental Table S1).

To screen other unknown genes regulated anthocyanin biosynthesis, we did the analysis of gene annotation and prediction of potential function. It is interesting to identify a candidate gene *Pbr006215.1* in DEGs, which shared 43.40% sequence identity with AtKAN4 [52]. The *Arabidopsis KAN4* was reported involving in ovule integument development and in modulating flavonol and proanthocyanidin content in seeds [53], however, it remains unclear whether *KAN4* is capable of regulating the biosynthesis of anthocyanin. Our data showed that *Pbr006215.1* expression was significantly higher in HNG than in NG (Log_2_ fold change = 1.84, *P* = 0.0072). A phylogenetic tree showed that there were three *KANADI* subfamilies in pear, and the *Pbr006215.1* was clustered with *AtKAN4* (Supplemental Fig. S1), we therefore assigned *Pbr006215.1* the name *PuKAN4*. To further identify the encoded protein, BLAST searches were conducted against the TAIR and NCBI databases, and we found that the CDS of *PuKAN4* was 996-bp-long with a 332-amino-acid protein, which included a GARP (*Golden2*, *ARR-B*, *Psr1)* domain (Supplemental Fig. S2).

Subsequently, we carried out RT-qPCR for appraising the mRNA abundance of the whole fruit development stage, and the findings also indicated lower *PuKAN4* expression in NG relative to HNG at 60, 90, 120, and 135 DAFB (Fig. 2G). These data suggest that *PuKAN4* may promote pear anthocyanin production.

To determine subcellular PuKAN4 localization, the leaves of *N. benthamiana* were transiently transfected with plasmids expressing GFP-tagged PuKAN4. As indicated by the findings shown in Supplemental Fig. S3, green fluorescence signal exhibited notable overlap with the NLS-mCherry nuclear marker, indicating the nuclear localization of PuKAN4. Therefore, a hypothesis of PuKAN4 being a TF was made.

### PuKAN4 represses anthocyanin biosynthesis

**Figure 3:**
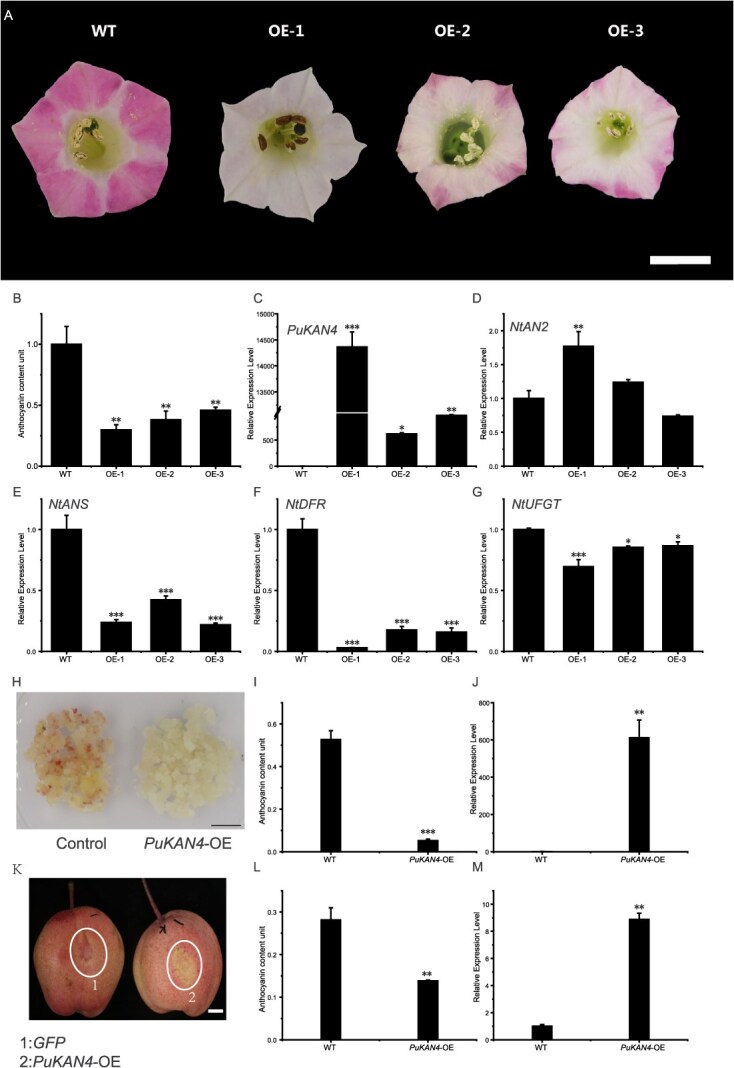
PuKAN4 suppressed anthocyanin accumulation in transgenic tobacco (*Nicotiana tabacum*), pear calli, and pear fruit. A: Phenotypic traits of control (WT) and *PuKAN4* overexpression (OE-1, 2, and 3) plants. B: Anthocyanin levels in *PuKAN4-*OE and WT tobacco flowers. C-G: Relative transcript levels of *PuKAN4, NtAN2, NtANS, NtDFR*, and *NtUFGT* in transgenic tobacco flowers as reflected by RT-qPCR. H: WT (control) and *PuKAN4*-OE pear calli phenotypic traits. I: Pear calli anthocyanin levels of different groups. J: Relative *PuKAN4* transcript abundance of different groups. K: Reduced anthocyanin biosynthesis in bagged ‘Hongzaosu’ fruits transiently transfected with a PuKAN4 OE plasmid. L: Skin anthocyanin levels of WT and PuKAN4 OE bagged ‘Hongzaosu’ fruits. M: Relative *PuKAN4* transcript abundance of control (GFP) and *PuKAN4*-OE bagged ‘Hongzaosu’ pear fruit skin. Scale = 1 cm for all panels. Error bars signify the mean ± SE of three biologically independent replicates. Two-tailed *t*-test *P*-values <0.001, 0.01, and 0.05 are, respectively, marked with ***, **, and *

*PuKAN4* expression was shown correlate with anthocyanin levels in HNG (*r* = 0.567, *P* = .028). We therefore next sought to verify whether PuKAN4 would affect anthocyanin regulation in transformed *N. tabacum*. The phenotypes observed in *N. tabacum* lines overexpressing *PuKAN4* were inconsistent with our hypothesis of PuKAN4 promoting anthocyanin generation. Specifically, the *PuKAN4*-overexpressing tobacco lines possessed white or light pink petals, whereas wild-type (WT) tobacco petals were red (Fig. 3A). The anthocyanin content was significantly lower in *PuKAN4*-overexpressing petals relative to the control (Fig. 3B), matching the observed phenotypes. Furthermore, RT-qPCR confirmed that *PuKAN4* was expressed at significantly higher levels in transgenic tobacco, while there was no expression in WT tobacco (Fig. 3C). The same results were obtained with semi-quantitative RT-PCR (Supplemental Fig. S4). Genes encoding the anthocyanin biosynthesis-related enzymes, namely *NtUFGT*, *NtANS*, and *NtDFR*, displayed evidently reduced expression in the *PuKAN4*-overexpressing petals (Fig. 3E-G). However, *NtAN2*, which encodes a crucial R2R3-MYB TF, displayed similar or greater transcript abundance comparing to WT, but not diminished expression after PuKAN4 overexpression (Fig. 3D). Collectively, it can be inferred on the basis of our data that PuKAN4 can suppress anthocyanin accumulation, possibly through repression of *NtANS*, *NtDFR*, and *NtUFGT*.

Next, the mechanisms responsible for PuKAN4’s suppressive effects on pear anthocyanin biosynthesis were explored in pear calli transformed with a *Cauliflower mosaic virus* (CaMV) 35S promoter-driven PuKAN4 overexpression plasmid. High light stimulated WT calli to turn red, whereas no such induction was observed in *PuKAN4*-overexpressing calli (Fig. 3H). WT calli also showed higher anthocyanin levels than *PuKAN4*-overexpressing calli (Fig. 3I). *PcANS*, *PcDFR*, and *PcUFGT* transcript abundances markedly diminished by PuKAN4 overexpression. However, *PcMYB10* and *PcMYB114* did not display evident transcript abundance variation between *PuKAN4*-overexpressing and WT calli (Supplemental Fig. S5). Subsequently, to further investigate PuKAN4’s role in regulating anthocyanin biosynthesis of pear, we constructed a 35S::PuKAN4 transient transformation model (with a 35S::GFP control) utilizing bagged ‘Hongzaosu’ fruits without anthocyanin reserves and applied strong light exposure to the fruits. Following the five-day light exposure, control fruits manifested red skin color, while the injection site of the pear skin showed a faint color in the PuKAN4 transiently overexpression fruit (Fig. 3K). Furthermore, the anthocyanin content in the infection site of control was higher than that of PuKAN4 transient overexpression (Fig. 3L). The successful overexpression of *PuKAN4* at the infection site is verified by the results displayed in Fig. 3M. The transcript abundances of *PpMYB10, PpDFR, PpANS*, and *PpUFGT* in the infection site of PuKAN4 overexpression were lower than that of the control, whereas that of *PpMYB114* was similar between the two groups (Supplemental Fig. S6). All these results supported that *PuKAN4* represses pear anthocyanin biosynthesis.

### PuKAN4 repressed anthocyanin pathway genes via the complex of PuMYB10/114-PubHLH3 

**Figure 4:**
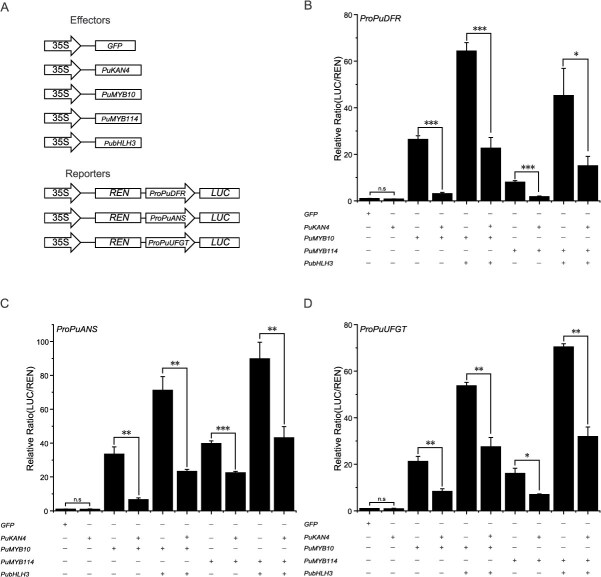
The PuKAN4-MYB complex represses expression of genes implicated with anthocyanin biogenesis. A: Plasmids constructed for dual luciferase experiments. B-D: Relative transcription activities of *ProPuANS*, *ProPuDFR*, and *ProPuUFGT* in *N. benthamiana* leaves as reflected by dual luciferase experiments. ‘+’ indicates addition; ‘-’ indicates no addition. Error bars represent the mean ± SE of four biologically independent replicates. Two-tailed *t*-test *P*-values <0.001, 0.01, and 0.05 are, respectively, designated ***, **, and *

Since PuKAN4 might impede the anthocyanin biosynthesis in pear fruit skin, pear calli, and *N. tabacum* by down-regulating LBGs, we further carried out dual-luciferase assays to explore whether PuKAN4 could directly regulate LBGs in tobacco. *LUC* reporter genes were driven by the promoter sequences of pear *PuDFR*, *PuANS,* and *PuUFGT*, with the expression of CaMV35S-driven *REN* (fused into the same cassette as test promoter fragments) utilized for data normalization. *GFP, PuKAN4*, *PuMYB10*, *PuMYB114*, and *PubHLH3* were driven by CaMV35S as effectors (Fig. 4A). Results showed that *PuKAN4* alone could not stimulate the activity of the *PuDFR*, *PuANS*, or *PuUFGT* promoters relative to 35S::GFP (Fig. 4B-D). Previous research has revealed that R2R3-MYB can partner with bHLH to exert crucial co-regulatory effects on the anthocyanin biosynthesis in plants. We therefore speculated that PuKAN4 may interact with an MYB to repress LBGs (*PuDFR*, *PuANS*, and *PuUFGT*). Our results showed that PuMYB10 and PuMYB114 could induce *PuDFR*, *PuANS*, and *PuUFGT* expression. However, the transactivation activity on *PuDFR*, *PuANS*, and *PuUFGT* significantly decreased when *PuMYB10* or *PuMYB114* was co-infiltrated with *PuKAN4* (Fig. 4B-D). *PubHLH3* is recognized as an important co-factor for PuMYB10/114 to activate expression of *PuDFR*, *PuANS*, and *PuUFGT* and thus promote anthocyanin accumulation in pear. Our results were consistent: *PuMYB10* or *PuMYB114* co-transformed with *PubHLH3* could sharply enhance the transactivation activity of the *PuDFR*, *PuANS*, or *PuUFGT* promoter compared to *PuMYB10* or *PuMYB114* alone. However, the promoter transactivation for *PuDFR*, *PuANS*, and *PuUFGT* sharply decreased when *PuKAN4* was added into the combination of *PuMYB10*/*114*-*PubHLH3*. These results indicated that PuKAN4 did not directly repress *PuDFR, PuANS*, or *PuUFGT,* but rather repressed the activating function of the PuMYB10/114-PubHLH3 complex, thus repressing transcription of *PuDFR, PuANS*, and *PuUFGT* in pear.

**Figure 5:**
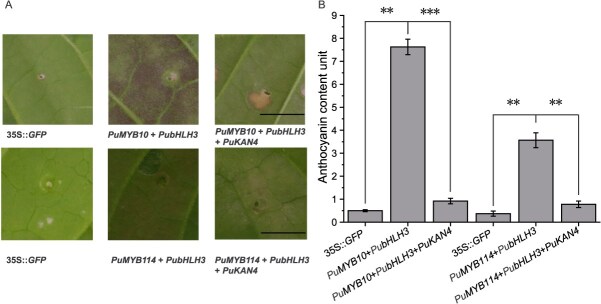
PuKAN4 via the PuMYB10/114-PubHLH3 to repress anthocyanin biosynthesis. A: *N. tabacum* leaves at five days after injection. PuMYB10/114 + PubHLH3 and 35S::GFP are, respectively, the positive and negative controls. Scale = 1 cm for all panels. B: Tobacco leaf anthocyanin abundances at five days after injection. Error bars denote the mean ± SE of three biologically independent replicates. Two-tailed *t*-test *P*-values <0.001 and 0.01 are, respectively, presented as *** and **

To demonstrate that PuKAN4 represses anthocyanin biosynthesis via the PuMYB10/114-PubHLH3 complex *in planta*, we transiently transformed these genes into *N. tabacum* leaves via infiltration. The genes were driven by CaMV35S (Fig. 4A). Our results showed that the leaves infiltrated with the *35S::GFP* negative control did not produce pigmentation after five days, whereas leaves co-infiltrated with *PuMYB10/PuMYB114* and *PubHLH3* exhibited red pigmentation. In contrast, little or no pigmentation was noted in leaves subjected to *PuMYB10/PuMYB114*, *PubHLH3*, and *PuKAN4* co-infiltration (Fig. 5A). The leaves co-infiltrated with *PuMYB10/PuMYB114* and *PubHLH3* were also found to have high anthocyanin accumulation, whereas anthocyanin accumulation was found to be low in leaves co-infiltrated with *PuMYB10*/*PuMYB114*, *PubHLH3*, and *PuKAN4* (Fig. 5B). These findings aligned well with the corresponding phenotypic observations and supported our previous hypothesis that PuKAN4 repressed anthocyanin biosynthesis by influencing the function of the PuMYB10/114-PubHLH3 complex.

### PuKAN4 interacts with PuMYB10 and PuMYB114

**Figure 6:**
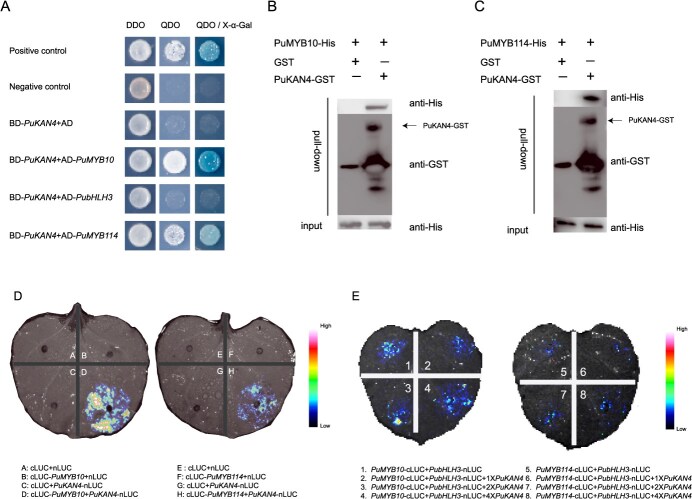
PuKAN4 interacts with PuMYB10 and PuMYB114 but not bHLH3.A: Y2H experiments showing interactions between PuKAN4 and PuMYB10/114 but not PubHLH3. X-α-gal: 5-bromo-4-chloro-3-indolyl-α-D-galactoside. DDO and QDO refer to double and quadruple dropout media lacking leucine+tryptophan and adenine+histidine+leucine+tryptophan, respectively. GST pull-down (B and C) and LCA (D) results verifying that PuKAN4 could interact with PuMYB10/114 in tobacco leaves

The interactions of PuKAN4 with PuMYB10, PuMYB114, and PubHLH3 were investigated via yeast two-hybrid experiments (Y2H). The result showed PuMYB10 and PuMYB114 exhibited robust interactions with PuKAN4, whereas no interaction was detected between PuKAN4 and PubHLH3 (Fig. 6A). These observed protein interactions were subsequently verified *in vitro* by glutathione S-transferase (GST) pull-down assays (Fig. 6B and C).

Luciferase complementation assays (LCA) were used to confirm the interaction between PuMYB114/PuMYB10 and PuKAN4. Co-expressing both cLUC-PuMYB114 + PuKAN4-nLUC and cLUC-PuMYB10 + PuKAN4-nLUC constructs in tobacco leaves produced an LUC signal, whereas the co-expression systems involving nonfused luciferase fragments (cLUC or nLUC) yielded no such signal (Fig. 6D), further validating the direct interactions between PuKAN4 and PuMYB10/114. Previous reports have shown that some repressors could bind with R2R3-MYB to hinder its interaction with bHLH [27]. We, therefore, hypothesized that interactions between PuKAN4 and PuMYB10/PuMYB114 may interfere with the formation of the MYB-bHLH complex. Co-infiltration of different concentrations of PuKAN4 with PuMYB10/PuMYB114 and PubHLH3 into *N. benthamiana* leaves were used to test this hypothesis. We found that the LUC signal changed very little or not at all as levels of *PuKAN4* were varied (Fig. 6E). This suggested that PuKAN4 did not interfere with MYB–bHLH interaction. Collectively, these data confirmed that PuKAN4 could directly interact with PuMYB10/PuMYB114, but not with bHLH3, and that the appearance of PuKAN4 did not prevent the formation of the PuMYB10/114-PubHLH complex. We thus infer that PuKAN4 might inhibit anthocyanin biosynthesis through the complex PuKAN4-PuMYB10/114-PubHLH3.

### PuKAN4 could repress anthocyanin biosynthesis through its conserved EAR motif

**Figure 7:**
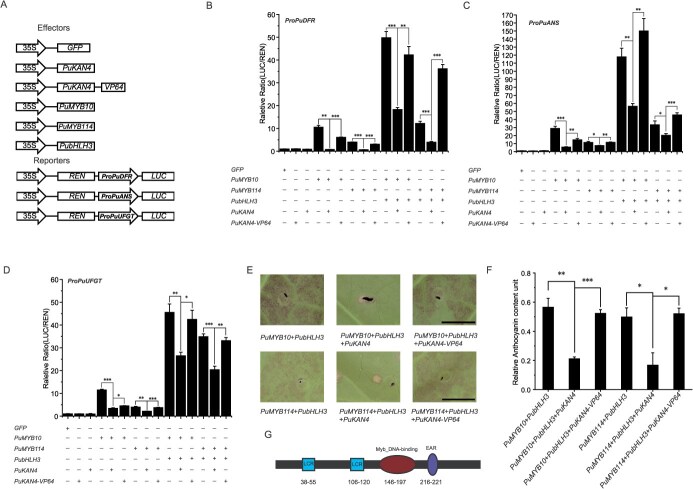
PuKAN4 actively repressed the PuMYB10/PuMYB114-PubHLH3 complex. A: Plasmids constructed for dual luciferase experiments. B-D: The transcriptional repression activity of PuKAN4 was inhibited by the VP64 transcription activation domain on the *PuDFR*, *PuANS*, and *PuUFGT* promoters. ‘+’ indicates addition; ‘-’ indicates no addition. E: Tobacco leaf appearances at five days after injection. *PuMYB10/114*-*PubHLH3* was the positive control. F: Anthocyanin levels in tobacco leaves at five days after injection. G: Predicted PuKAN4 secondary structure. Error bars stand for the mean ± SE of three (for anthocyanin content assays) or four (for dual luciferase assays) biologically independent replicates. Scale = 1 cm for all panels. Two-tailed *t*-test *P*-values <0.001, 0.01, and 0.05 are, respectively, symbolized by ***, **, and *

Next, we tested if PuKAN4 could directly occupy promoters of anthocyanin structural genes in an MYB–bHLH independent manner. We performed dual luciferase assays using a fusion of *PuKAN4* with *VP64*, which is widely reported as a transcription activation domain [54]. The reporters and effectors were constructed as described in Fig. 7A. Only transformation with the *35S::PuKAN4-VP64* construct showed no activation of the *PuDFR*, *PuANS*, and *PuUFGT* promoters, comparable to the results seen with the empty vector control (Fig. 7B-D). Co-infiltration of *PuMYB10/PuMYB114* or with *PubHLH3* showed strong activation of the *PuDFR*, *PuANS*, and *PuUFGT* promoters, and again the transactivation activity dramatically decreased when *PuMYB10/PuMYB114* and *PubHLH3* were co-infiltrated with *PuKAN4* (Fig. 7B-D). However, the transactivation activity was significantly higher when *PuMYB10/PuMYB114* and *PubHLH3* were co-infiltrated with *PuKAN4-VP64* (Fig. 7B-D). These results imply that PuKAN4 cannot directly regulate *PuDFR*, *PuANS*, and *PuUFGT*, and PuKAN4 is an active suppressor.

To verify the mechanism of PuKAN4 repressing the anthocyanin biosynthesis through active repression, the transient transformation of tobacco leaves were used. The red color in tobacco leaves recovered when the PuKAN4-VP64 was transformed in combination with PuMYB10/MYB114 and PubHLH3 (Fig. 7E and F), which indicated that the repression function of PuKAN4 could be offset by VP64. To exclude the direct effects of PuKAN4-VP64 on anthocyanin biosynthesis, we conducted transient overexpression of GFP, PuKAN4-VP64, PuKAN4, PuMYB10, PuMYB114, PuMYB114 + PuKAN4-VP64, and PuMYB10 + PuKAN4-VP64, but the red color or anthocyanin content were not detected in all these tobacco leaves (Supplemental Figs S7 and S8), which indicted that PuKAN4-VP64 could not affect the anthocyanin biosynthesis directly. These results proved that PuKAN4 could repress anthocyanin biosynthesis, which interacted with PuMYB10/MYB114 and did not affect the combination of PuMYB10/MYB114-PubHLH3, suggesting that PuKAN4 was an active suppressor and formed PuKAN4-PuMYB10/PuMYB114-PubHLH3 complex to repress anthocyanin biosynthesis.

PuKAN4 secondary structure was subsequently predicted with the SMART tool [55]. There were two low complexity regions (LCRs), which were located at residues 38 to 55 and at 106 to 120; there was also an MYB DNA binding site located at residues 146 to 197 (Fig. 7G). Importantly, an ethylene-responsive element-binding factor-associated amphiphilic repression motif (EAR) motif (DLNQNP) that frequently exerts suppressive effects on gene transcription [56] was found at residues 216 to 221. This further suggested that PuKAN4 was an active repressor of anthocyanin biosynthesis. We also compared KAN4 protein sequences between *Arabidopsi*s and species of the family *Rosaceae.* KAN4s contained a conserved GARP domain and conserved motifs at amino acid positions 9 to 21 and 306 to 318 (Supplemental Fig. S2). Although the KAN4 proteins investigated are orthologues and shared similar protein sequences, only PuKAN4 in pear and MdKAN4 in apple had the EAR inhibitory motif.

### *PuKAN4* transcription was activated by PuMYB10 and PuMYB114, creating a regulatory feedback loop

**Figure 8:**
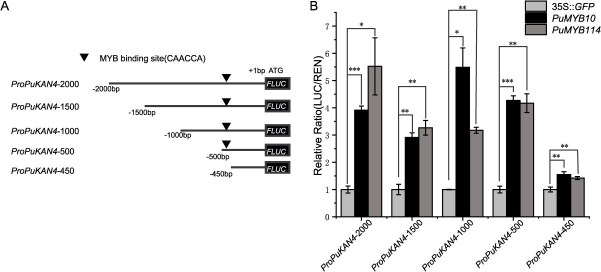
PuMYB10/114 activates *PuKAN4* transcription by binding to its promoter. A: Graphic representation displaying all analyzed *PuKAN4* promoter fragments (including the 2 kb promoter region and three truncated promoters). The triangle indicates the MYB binding motif (CAACCA). B: Transcription activities of the 2-kb and three truncated *PuKAN4* promoters upon the overexpression of PuMYB10/114. Relative LUC/REN activity driven by the *PuKAN4* promoter upon transfection of the empty 35S::GFP plasmid was utilized for normalization of dual luciferase experiment data. Error bars denote mean ± SE of three biologically independent replicates. Two-tailed *t*-test *P*-values <0.001, 0.01, and 0.05 are, respectively, represented by ***, **, and *

The results presented above demonstrate that PuKAN4 interacted with the PuMYB10/PuMYB114-PubHLH3 complex to repress the LBGs *PuDFR, PuANS*, and *PuUFGT*. Previous research has elucidated the hierarchical regulation of activators and repressors involved in plant anthocyanin biosynthesis [22, 57, 58]. With this in mind, we further analyzed the *PuKAN4* promoter using PlantCARE (http://bioinformatics.psb.ugent.be/webtools/plantcare/html/) and identified a CAACCA motif, which could potentially be recognized by MYB, at −460 to −454 bp relative to *PuKAN4* coding region (Fig. 8A). Based on these results, we hypothesized that *PuKAN4* can be activated by PuMYB10 and PuMYB114 and carried out dual-luciferase experiments to test this hypothesis. The findings showed that PuMYB10/114 could activate a 2-kb promoter fragment immediately preceding the *PuKAN4* coding sequence (Fig. 8B). Then this 2-kb promoter region was truncated into 1.5-kb, 1-kb, 500-bp, and 450-bp fragments immediately preceding the *PuKAN4* coding sequence to pinpoint the PuMYB10/114-binding site (Fig. 8A). PuMYB10/PuMYB114 could strongly activate the 1.5-kb, 1-kb, and 500-bp *PuKAN4* promoter fragments (Fig. 8B). However, the LUC/REN ratio was much lower in the samples containing the 450-bp promoter fragment compared to the 1.5, 1, or 500-bp fragments or the entire 2-kb promoter region, as the 450-bp promoter fragment did not contain the MYB binding site (CAACCA). These results indicated that PuMYB10/PuMYB114 activated *PuKAN4* expression through direct binding to its promoter. Dual-luciferase assays were also implemented to verify that when PuMYB10/PuMYB114 activates *ProPuKAN4* to promote *PuKAN4* expression, expression of anthocyanin-related genes is repressed. *ProPuDFR*, *ProPuANS*, and *ProPuUFGT* are strongly activated by PuMYB10/PuMYB114; however, when PuMYB10/PuMYB114 were co-infiltrated with *ProPuKAN4::PuKAN4*, the activation effects of PuMYB10/114 on these LBGs decrease significantly (Supplemental Fig. S9). This result also indicated that *PuMYB10/PuMYB114* can activate *PuKAN4* expression to subsequently inhibit anthocyanin-related LBGs.

## Discussion

### *PuKAN4* inhibits pear anthocyanin biosynthesis

Our group previously reported that PuMYB10 and PuMYB114 activate LBGs to enhance pear anthocyanin production [12]. Herein, an inhibitory effect of *PuKAN4* on the biosynthesis of plant anthocyanin was characterized. Recently, a diverse group of repressors for plant anthocyanin biosynthesis have been characterized, including *SlMYBATV* [59], *PpBBX21* [60], *AtSPL9* [39], *PpCOP1* [61], and *MdHB1* [38]. Overexpression of *PuKAN4* in pear calli and tobacco plants demonstrated that it could repress the biosynthesis of anthocyanin. Similarly, overexpression of apple HD-Zip I transcription factor *HB1* in tobacco reduces petal anthocyanin accumulation, functioning as a repressor [38]. Overexpression of *GmMYBR* also decreases anthocyanin accumulation in soybean flowers and hypocotyls by functioning as a repressor[62].

MYBs are the most commonly known repressors of anthocyanin biosynthesis and comprise two phylogenetic clades: subgroup 4 R2R3-MYBs and R3-MYBs [63, 64]. Some reports have shown that SPLs, HD-ZIPs, and miRNAs can also act as repressors of anthocyanin biosynthesis [24]. The *GARP* transcription factor family of genes functions in many processes in plants, including signal transduction, transportation of hormones, development of chloroplast, shoot and root, floral transition, and circadian clock oscillation maintenance [65]. *KAN4* is a member of the *KANADI* subfamily in the *GARP* gene family in *Arabidopsis* [66]. *KANADI* was reported to regulate plant ovule integument development, adaxial–abaxial polarity, and gibberellic acid (GA) and auxin signaling [52, 65–67]. OsKAN1 directly binds to the *OsYABBY5* (*OsYAB5*) promoter to repress its expression and interacts with OsYAB5 to form a functional OsKAN1–OsYAB5 complex, which inhibits OsYAB5-induced *OsGA2ox6* expression [68]. *AtKAN4* of *Arabidopsis* regulates seed levels of flavonols and proanthocyanidins (PAs) [53]. However, the *KANADI* subfamily has rarely been implicated with anthocyanin biosynthesis regulation.

### PuKAN4 interacts with the PuMYB10/PuMYB114-PubHLH3 to repress anthocyanin biosynthesis

Members of the GARP family have for some time been considered as MYB or MYB-like transcription factors because their B-motif is similar to the MYB/MYB-like domain. Members of the GARP family have now been further classified [65]. PuKAN4 is a KANADI subfamily protein that does not contain a [D/E]LX_2_[R/K]X_3_LX_6_LX_3_R bHLH-interacting motif. Our study confirmed with a Y2H assay that PuKAN4 does not interact with bHLH3, although it looks like a MYB-like domain-containing protein. *PuKAN4* encodes a conserved EAR repression motif, which could recruit Groucho (Gro)/Tup1 to exert chromatin modification and transcription inhibition effects [69]. However, no direct inhibitory effects of PuKAN4 towards genes participating in anthocyanin biosynthesis were observed.

Some transcription factors negatively regulate MYB activators to repress anthocyanin biosynthesis. For example, through competing with PpBBX18 for interaction with PpHY5, PpBBX21 interferes with the PpHY5–PpBBX18 interaction, thereby reducing *PpMYB10* expression and repressing pear anthocyanin production [60]. When expressed in *Arabidopsis*, *MYB4* may suppress *TT2*, *MYB90*, and *MYB75* expression in an indirect manner to reduce anthocyanin accumulation [70]. Herein, the transcript abundances of activators *PuMYB10/PuMYB114* were not affected in tobacco or pear calli when *PuKAN4* was overexpressed. These results indicated that PuKAN4 did not repress the expression of activator MYBs to suppress anthocyanin biosynthesis.

As a protein complex with high conservativeness, MYB-bHLH-WDR (MBW) serves to activate plant anthocyanin biosynthesis and its formation can be impeded by several repressors [24]. For example, many MYB repressors have a bHLH-interacting motif, which competes for bHLH binding with MYB activators, such as tomato *MYBATV* [36], monkeyflower *RTO* [34], and petunia *MYBx* [27]. Additionally, some MYB proteins possess an R3-MYB domain motif that interacts with bHLH and a unique C-terminal repression motif (a TLLLFR and/or EAR motif), both of which compete for bHLH binding with MYB activators; these motifs may also show repressive activity via a unique repression motif, such those seen in apple *MYB16* [71], poplar *MYB182* [28], soybean *MYBR* [62], and peach *MYB18* [29]. SPL9 can repress *Arabidopsis* anthocyanin production via separating MYB from bHLH, destabilizing the MBW transcription-activating heterotrimer [39]. HAT1 can bind to MYB75, interfering with the MBW protein complex [72]. MdHB1 can bind with MdMYB10, MdbHLH3, and MdTTG1, impeding the nuclear translocation of MBW complex and thus suppressing *MdUFGT* and *MdDFR* transcription in an indirect manner in apple [38]. PuKAN4 can directly interact with PuMYB10/PuMYB114, but not with bHLH3, and the PuKAN4 did not prevent the formation of the PuMYB10/114-PubHLH3 complex (Figs 6, 7G, and S5). PuKAN4 appears to neither remove nor disrupt the MYB-bHLH protein complex, but does interact with it to repress anthocyanin biosynthesis through the EAR motif of PuKAN4.

### PuKAN4 may balance anthocyanin biosynthesis to avoid over-accumulation in pear

We identified a binding element for MYB (CAACCA) within *PuKAN4* promoter and hypothesized that *PuKAN4* may be regulated by PuMYB10 and PuMYB114 in pear. It has previously been reported that R2R3 MYBs bind to the CAACCA consensus sequence to regulate target gene transcription, such as that of *Brachypodium distachyon MYB41* [73] and peach *MYB10.1* [4]. According to our RT-qPCR results, *PuKAN4*, *PuMYB10*, and *PuMYB114* were found to be more highly expressed in HNG than in NG. We also confirmed via dual luciferase assays that PuMYB10/PuMYB114 activated *PuKAN4* expression. These findings suggested that *PuKAN4* was activated by PuMYB10/PuMYB114 in pear, forming a feedback loop to regulate anthocyanin biosynthesis. Similarly, *FaMYB1*, an anthocyanin repressor in strawberry, is also highly expressed when there are high levels of anthocyanin accumulation, balancing anthocyanin levels [26]. The anthocyanin repressor *PhMYBx* is expressed during anthocyanin accumulation in petunia, which may produce feedback repression [74]. A high abundance of PpMYB18 is essential for maintaining a balance between the accumulative amounts of proanthocyanidin and anthocyanin within red-fleshed peach. [29]. In tomato, the critical activator Aft can target the promoter of *MYBATV*, an anthocyanin production repressor, to promote *MYBATV* expression in transgenic tomato [36]. *RTO*, an anthocyanin synthesis inhibitor, is activated by NEGAN, and these two transcription factors form a feedback loop to modulate monkeyflower anthocyanin biosynthesis [34]. *GmMYBR* is a repressor that is activated by GmMYBA2, and the two may form a feedback loop that regulates soybean seed coat coloration[62].

Feedback regulatory mechanisms exist within many metabolic pathways in plants, such as ethylene biosynthesis [75, 76], wood formation [77] and reactive oxygen species production [78]. Feedback regulation may provide biological robustness to protect internal cellular and organismal stability [79–83]. Anthocyanin production regulation involves many stimulatory and inhibitory factors to enable anthocyanin accumulation at the correct spatial locations and at the appropriate concentrations [27]. Anthocyanins function in multiple processes, including antioxidation, UV damage prevention, pollinator attraction, and seed dispersal [84–86]. Nevertheless, excessive anthocyanin could undermine the balance between general and specialized metabolites [87]. For example, anthocyanin accumulation may induce starch degradation in purple sweet potato and affect starch structure in Thai pigmented rice [88, 89]. Anthocyanins compete for phenylpropanoid metabolites derived from lignin biosynthesis that would otherwise promote cell wall thickness in secondary-thickened cells [90, 91]. Excessive anthocyanin may not only cause metabolite imbalances, but also may affect the development of plant organs [92, 93]. Apple anthocyanin over-accumulation can also drive internal browning disorders [94, 95].

In this study, we demonstrated that the expression of *PuKAN4* is positively regulated by *PuMYB10* and *PuMYB114*, confirming the existence of a feedback regulatory mechanism in anthocyanin biosynthesis. Previous studies have shown that anthocyanin biosynthesis can persist during the night in pear fruit skins [96], despite being typically influenced by environmental factors such as light [97] and temperature [98]. We hypothesize that the expression levels of *PuMYB10* and *PuMYB114* may decline in the afternoon or evening, even as anthocyanin biosynthesis continues. During this period, *PuKAN4* may remain expressed under conditions of low temperature, reduced light, or darkness, potentially acting as a suppressor to fine-tune anthocyanin biosynthesis. This regulatory mechanism could serve to conserve energy and prevent the excessive accumulation of anthocyanins, thereby maintaining a balance between biosynthetic activity and metabolic demands.

**Figure 9:**
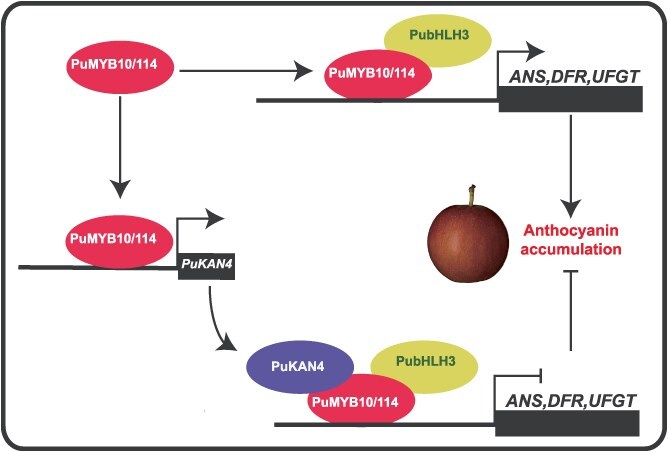
*PuKAN4*’s role in regulating anthocyanin biosynthesis. We propose a model in which a PuKAN4-PuMYB10/114-PubHLH3 feedback loop regulates anthocyanin biosynthesis to ensure fine-tuning of its accumulation in pear. Expression of *PuMYB10* and *PuMYB114* are induced by light, which, together with PubHLH3 as a co-regulator, activate anthocyanin structural genes. *PuKAN4* expression can be induced by *PuMYB10*/*114* and can repress the function of the MYBs–bHLH3 complex. These results revealed that an activator-and-repressor feedback loop was formed between PuMYB114/PuMYB10 and PuKAN4, which possibly to balance biosynthesis activity and prevent over-accumulation of phenylpropanoids

Taken together, on the basis of the findings of this research, we propose a model in which a PuKAN4-PuMYB10/114-PubHLH3 feedback loop regulates anthocyanin biosynthesis to ensure fine-tuning of its accumulation in pear. The expression of *PuMYB10* and *PuMYB114* are induced by light, which, together with PubHLH3 as a co-regulator, activate anthocyanin structural genes. *PuKAN4* expression can be induced by *PuMYB10*/*114* and can repress the function of the MYBs-bHLH3 complex. These results revealed that an activator-and-repressor feedback loop was formed between PuMYB114/PuMYB10 and PuKAN4, which possibly to balance biosynthesis activity and prevent over-accumulation of phenylpropanoids (Fig. 9).

## Materials and methods

### Collection and preparation of plant materials

Two pear (*Pyrus ussuriensis*) cultivars were utilized for this research: ‘Nanguo’ (NG), which had a green or slightly red peel, and ‘Hongnanguo’ (HNG), which had a deep red peel. The fruit samples were collected from Haicheng, Liaoning Province, China and were harvested on 30, 60, 90, 120, and 135 days after full bloom (DAFB). Five fruits were gathered to constitute a biological replicate, with three replicates collected at each stage during plant development. The peels were harvested from the collected fruits, snap frozen with liquid nitrogen and kept under −80°C.

### Isolation of total RNA and RT-qPCR assays

The samples were subjected to RNA extraction employing a Plant Total RNA Isolation Kit Plus (Foregene, China), followed by synthesis of cDNA utilizing HiScript II Q RT SuperMix for qPCR (+gDNA wiper) (Vazyme, China) as per the provided protocol. The FastStart Universal SYBR Green Master kit (Roche, Switzerland) was adopted for the establishment of RT-qPCR reaction system, while the thermal program was implemented with the Roche LC480 instrument. Pear t*ubulin* [99] and tobacco *NtEF1α* [100] were used as internal references for data normalization. The 2^-ΔΔCt^ algorithm was employed for determining relative target transcript abundance [101]. There were three biologically independent repeats for each experiment. Supplemental Table S2 summarizes the primers.

### Isolation and quantification of anthocyanin

The protocol of Yao *et al.* [12] was employed for extracting anthocyanin. Briefly, fruit peel (pear) or petals/leaves (tobacco) were ground into powder using liquid nitrogen, which was subjected to a 12-h incubation at 4°C in cold methanol containing 1‰ HCl, and the mixture was subjected to a 20-min centrifugation at 12 000 *g*, 4°C. The liquid phase was then analyzed by UV–vis spectrophotometry for 530-, 620- and 650-nm optical density values. The equation shown below was adopted for calculating anthocyanin content:

$\left[\left(\mathrm{A}530-\mathrm{A}620\right)-0.1\times \left(\mathrm{A}650-\mathrm{A}620\right)\right]/\mathrm{fresh}\ \mathrm{weight}.$

For each treatment or cultivar, three biologically independent samples were subjected to this analysis.

### RNA-seq

NG and HNG fruit peel samples collected at 135 DAFB were utilized for RNA-seq. Novogene (Beijing, China) conducted the isolation of total RNA and construction of sequencing libraries. Paired-end reads (125 bp in length) were generated by RNA-seq performed with Illumina HiSeq 2500. By adopting cut-off thresholds of fold change <0.5 or > 2.0 and *P*-value ≤0.05, DEGs were determined. A gene’s fragments per kilobase per million mapped reads values of HNG and NG groups were computed for calculating fold changes. Three biologically independent replicates of each cultivar were analyzed.

### Tobacco transformation

Tobacco transformations were implemented in accordance with a published strategy [102]. For transformation of the *35S::PuKAN4*-expressing *Agrobacterium* GV3101 strain into tobacco leaves, the leaf disc strategy was adopted*.* Leaf discs were placed on medium with kanamycin (150 mg/L) and timentin to screen for transformants that could regenerate shoots. The regenerated shoots were subsequently induced into roots. Kanamycin selection and qPCR were utilized for transformed plant screening. Blooming tobacco flowers were imaged, then snap frozen with liquid nitrogen and kept under −80°C.

### Pear calli transformation

For transformation of pear calli, we adopted a published strategy; juvenile fruit tissues of the pear variety Clapp’s Favorite were employed to induce the formation of calli [103]. *Agrobacterium* strain EHA105 was transformed with pCAMBIA1301-PuKAN4 recombinant plasmids via electroporation. Pear calli were immersed in *Agrobacterium* culture (OD_600_ = 0.4) and shaken at ambient temperature, 120 rpm for 20 min. After drying, pear calli were co-cultured for 2 days on MS screening medium supplemented with cefalotin and hygromycin at 20 mg/L, then incubated at 24°C under the continuous dark. The calli were transferred into a growth chamber with constant light exposure to receive light treatment.

### PuKAN4 subcellular localization

For establishment of the *35S::PuKAN4* construct, *PuKAN4* complete coding sequence was incorporated into the pCAMBIA1300 binary vector. Electrotransformation was subsequently carried out to introduce the empty and recombinant plasmids into *Agrobacterium* GV3101. The transformants were subjected to infiltration into nuclear-localized mCherry-expressing tobacco leaves. After 3 d, the leaves were analyzed via confocal microscopy (LSM 800, Zeiss, Germany). Supplemental Table S2 displays the primers synthesized for plasmid construction.

### Dual luciferase experiments

Transient expression and dual-luciferase experiments were carried out in accordance with the methods detailed by Liu *et al.* [104]. The coding sequences of *PuKAN4*, *PuMYB10*, *PuMYB114*, and *PubHLH3* were each inserted into pSAK277 [105]. *PuDFR*, *PuANS*, *PuUFGT*, and *PuKAN4* promoter fragments were each incorporated into the pGreenII 0800-LUC [105] vector. Electrotransformation was subsequently carried out to introduce the recombinants into GV3101 or GV3101 (with pSoup) to generate strains. An infiltration buffer (150 μM acetosyringone, 10 mM MgCl_2_, and 10 mM MES; pH 5.6) was employed to adjust the concentration of transformants into OD_600_ = 1.0. Subsequently, a needleless syringe was employed for infiltration of an *Agrobacterium* mixture containing (TFs: promoters = 9:1) into tobacco leaves. Seventy-two hours following *Agrobacterial* infiltration, activities of Renilla and firefly luciferases (LUC) were gauged as per protocols of providers (Promega, USA; Yeasen, China). The activity of LUC was normalized to that of REN to represent basal 35S promoter activity. All ratios of LUC/REN were normalized to that of the pSAK277 empty vector, which was assigned a value of 1. Each assay involved three or four biologically independent repeats. Supplemental Table S2 displays the primers utilized for these experiments.

### LCA

A strategy utilized in a published paper was adopted for LCA [106]. *PuKAN4* and *PubHLH3* were each inserted into a pCambi1300-nLUC vector and *PuMYB10* and *PuMYB114* were each introduced into a pCambi1300-cLUC vector. Afterwards, electrotransformation was implemented to introduce the empty and recombinant plasmids into GV3101, and the cLUC- and nLUC-expressing transformants were subjected to co-infiltration into tobacco leaves. At 2 d post infiltration, the leaves were further treated with D-luciferin (0.5 mM, Yeasen, China) and analyzed using a PIXIS 1024B (Princeton Instruments, Trenton, NJ, USA). The primers are detailed in Supplemental Table S2.

### Y2H assay

For Y2H experiments, a previously utilized protocol was followed [12]. The complete coding sequence of *PuKAN4* and those of *PuMYB10*, *PuMYB114*, and *PubHLH3* were, respectively, incorporated into pGBKT7 and pGADT7 vectors Co-transformation was implemented to introduce the recombinants into AH109 yeast strain. Double dropout and X-α-Gal-supplemented quadruple dropout media lacking leucine+tryptophan and adenine+histidine+leucine+tryptophan were, respectively, employed for transformant selection and protein interaction analysis. Yeast co-transformed with pGADT7-T and pGBKT7-Lam were the negative controls and those with pGADT7-T and pGBKT7–53 were the positive controls. Following a 3–5-day incubation at 30°C, the yeast culture plates were subjected to visual inspection. Supplemental Table S2 lists all the primers used for Y2H experiments.

### Pull-down assay

Pull-down assays were performed as described by Wei *et al.* [107]. The recombinant vectors constructed were PuMYB10 with a polyhistidine (His) tag (PuMYB10-His) (pCOLD-TF), PuMYB114-His (pCOLD-TF), and PuKAN4 with a glutathione S-transferase (GST) tag (PuKAN4-GST) (pGEX-4 T-1). The constructs were transformed into *Escherichia coli* strain Rosetta (DE3). For protein induction, *E. coli* was incubated at 37°C with shaking at 220 rpm. Isopropyl β-d-1-thiogalactopyranoside at 1 M was added to the *E. coli* culture to a final concentration of 1 mM; the OD_600_ was 0.6–0.8. The PuMYB10-His and PuMYB114-His cultures were induced for 16 h at 16°C, and the PuKAN4-GST culture for 16 h at 20°C. For the pull-down assay, GST or PuKAN4-GST and PuMYB10 or PuMYB114 were incubated in a binding buffer with GST Sefinose™ Resin (Sangon, Shanghai, China), then washed thoroughly. Proteins were then resolved via SDS-PAGE(1 610 183, BIO-RAD, USA) and analyzed with an immunoblot (CW2030M, CWBIO, China) using anti-GST(D190101, Sangon, Shanghai, China) and anti-His antibodies (D191001, Sangon, Shanghai, China). The primer sequences are listed in Supplemental Table S2.

### Transient color assay in *N. Tabacum* leaves

Transient expression assays in *N. tabacum* were carried out as previously described [108]. The CDSs of *PuKAN4*, *PuMYB10*, *PuMYB114*, and *PubHLH3* were inserted into the pSAK277 vector, and the plasmids were electroporated into *Agrobacterium* strain GV3101. Young tobacco leaves were infiltrated with *Agrobacterium*. Photos of the leaves were taken at 5 d postinoculation. Leaf samples were frozen in liquid nitrogen, and then stored at −80°C. The primer sequences are shown in Supplemental Table S2.

### Transient transformation of pear fruits

Bagged ‘Hongzaosu’ pear fruits near ripening were harvested for the transient transformation. The CDSs of *PuKAN4* were inserted into the pSAK277 vector for overexpression under the control of the 35S promoter. The recombinant plasmid was transformed into *Agrobacterium* strain GV3101. *Agrobacterium* were adjusted to OD_600_ = 1.0 with infiltration buffer (10 mM MES, 10 mM MgCl_2_, and 150 uM acetosyringone; pH 5.6) and were then used for infiltration. After infiltration, the pear fruits were incubated in a growth chamber under continuous white light at 25°C. The fruit phenotypes were observed and analyzed 5 days later, and the fruit peels were frozen in liquid nitrogen, then stored at −80°C for further experiments.

### Statistical analysis

In this study, statistical analysis was conducted using the SPSS 20.0 package for Windows (SPSS, Inc., Chicago, IL), and Student’s *t*-test was applied. All data were presented as mean ± standard error (SE). Statistical significance was considered at *P < 0.05, **P < 0.01, and ***P < 0.001.

## Gene and accession numbers

Sequencing data can be retrieved using the following GenBank accession numbers: *PuKAN4,* KAB2628042.1; *PubHLH3*, JX403960.1; *PuMYB10*, KF387520.1; *PuMYB114*, MF489219.1.

## Supplementary Material

Web_Material_uhaf071

## Data Availability

The data generated for this study have been deposited in the United States National Center for Biotechnology Information (NCBI) Sequence Read Archive (SRA) under the accession number PRJNA814481.
