# Supplementary figures and images for: Characterization of a KANADI-like transcription factor that suppresses pear anthocyanin biosynthesis
Source: Hortic Res. 2025 Mar 3;12(6):uhaf071. doi: 10.1093/hr/uhaf071 (PMC12038239; doi:10.1093/hr/uhaf071)

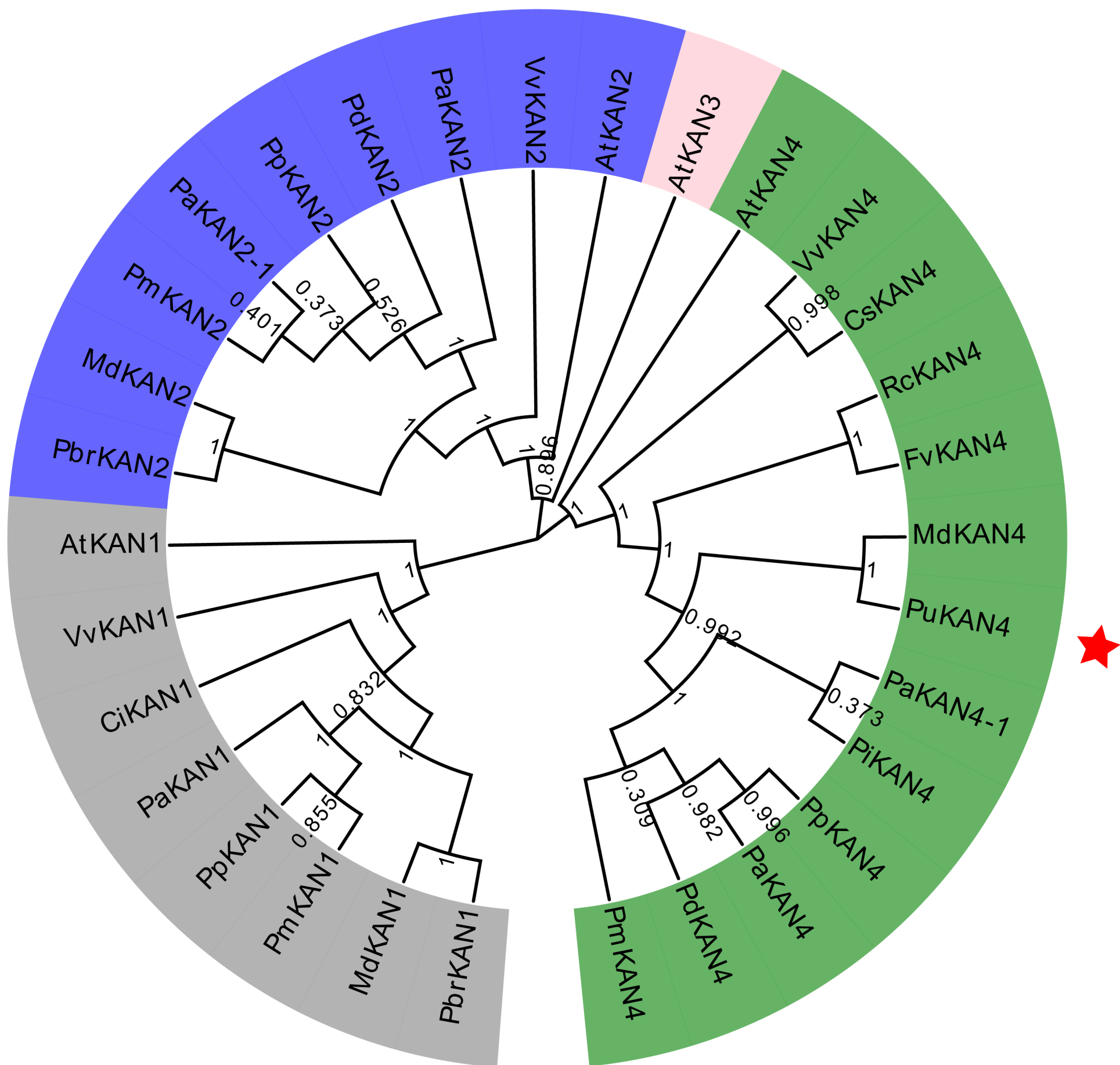

Supplement: Web_Material_uhaf071 [file web_material_uhaf071.zip › figure s1.pdf]

Merged

GFP

Bright field

mCherry

*PuKAN4-GFP*

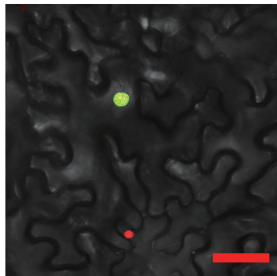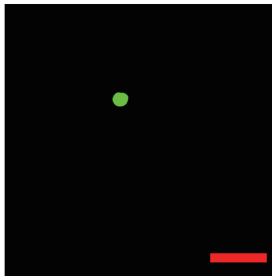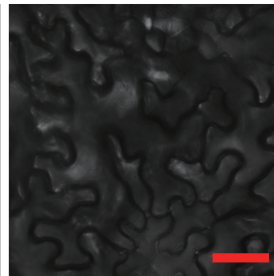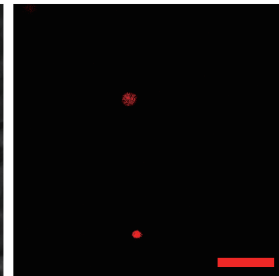

*35S-GFP*

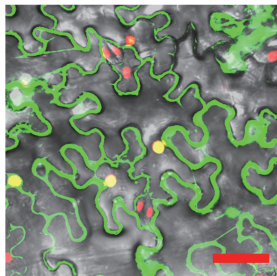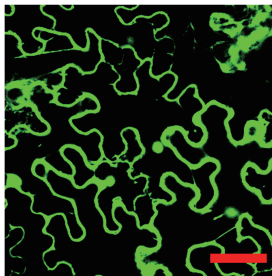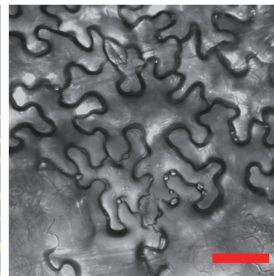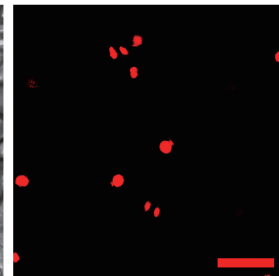

Supplement: Web_Material_uhaf071 [file web_material_uhaf071.zip › figure s3.pdf]

WT

OE-1

OE-2

OE-3

*PuKAN4*

*NtEF1a*

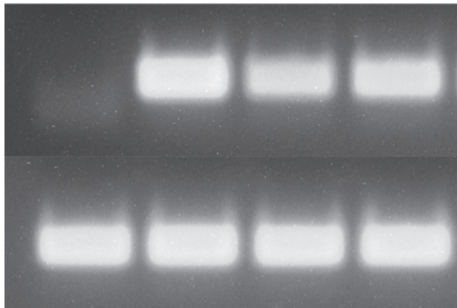

Supplement: Web_Material_uhaf071 [file web_material_uhaf071.zip › figure s4.pdf]

Relative expression level

2

1

0

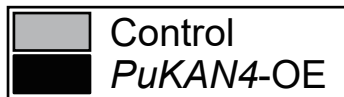

*PcMYB114*

*PcMYB10*

*PcDFR*

*PcANS*

*PcUGFT*

\*\*\*

\*\*\*

\*

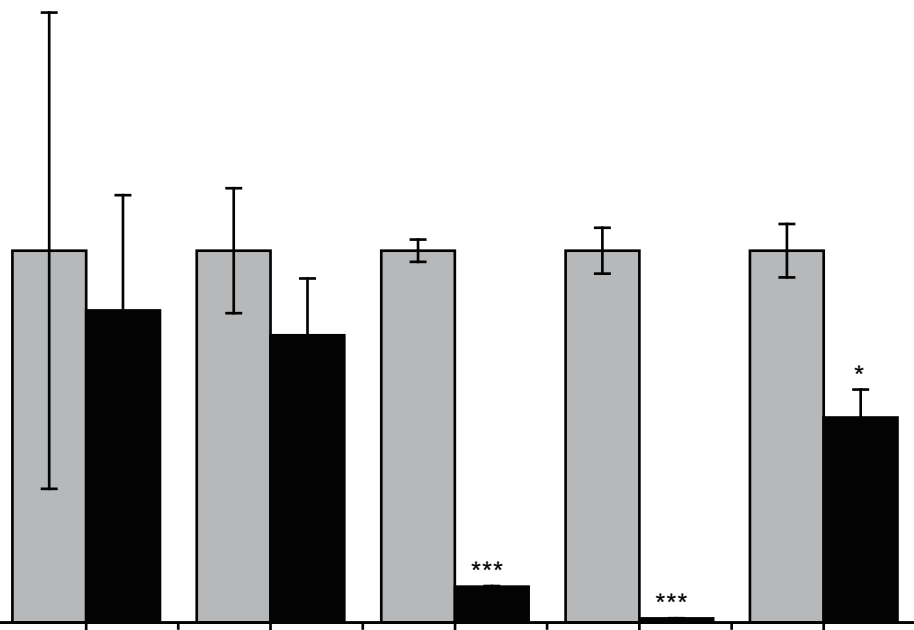

Supplement: Web_Material_uhaf071 [file web_material_uhaf071.zip › figure s5.pdf]

Relative expression level

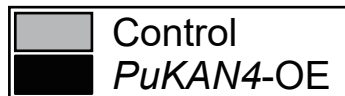

2  
1  
0

*PpMYB114*

*PpMYB10*

*PpDFR*

*PpANS*

*PpUFGT*

\*\*

\*

\*\*

\*\*

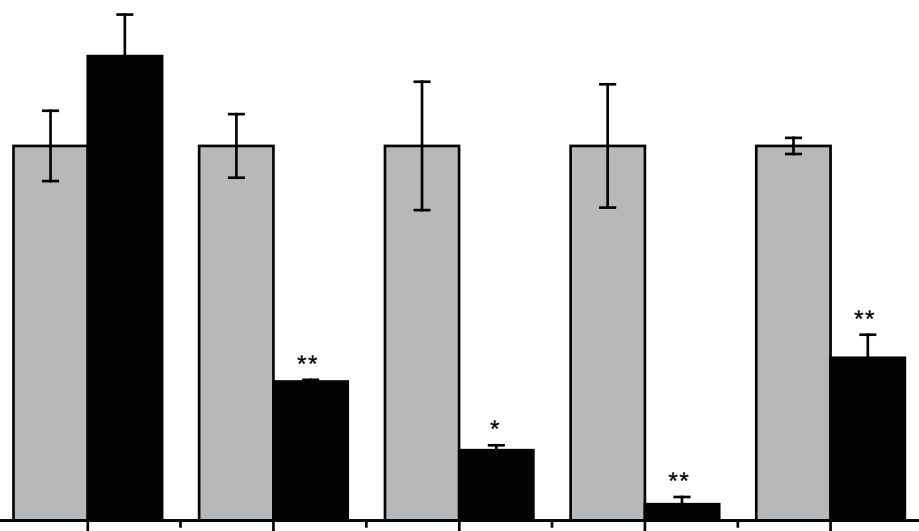

Supplement: Web_Material_uhaf071 [file web_material_uhaf071.zip › figure s6.pdf]

A

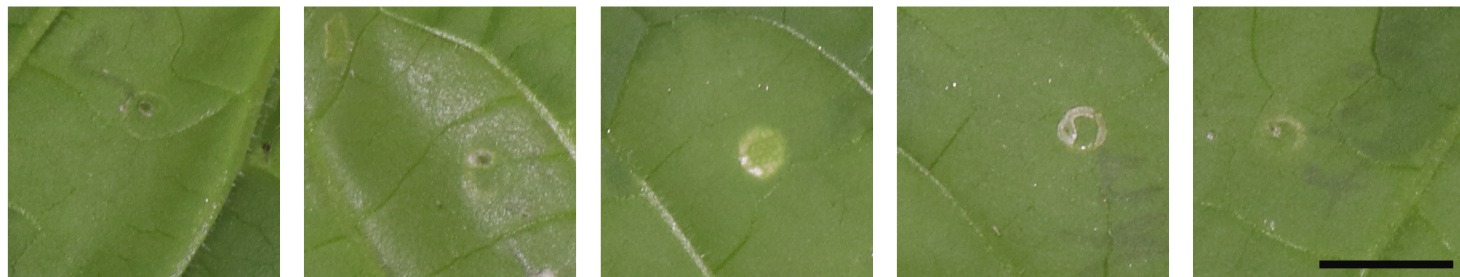*GFP**PuKAN4**PuMYB10**PuKAN4-VP64**PuMYB10+*  
*PuKAN4-VP64*

B

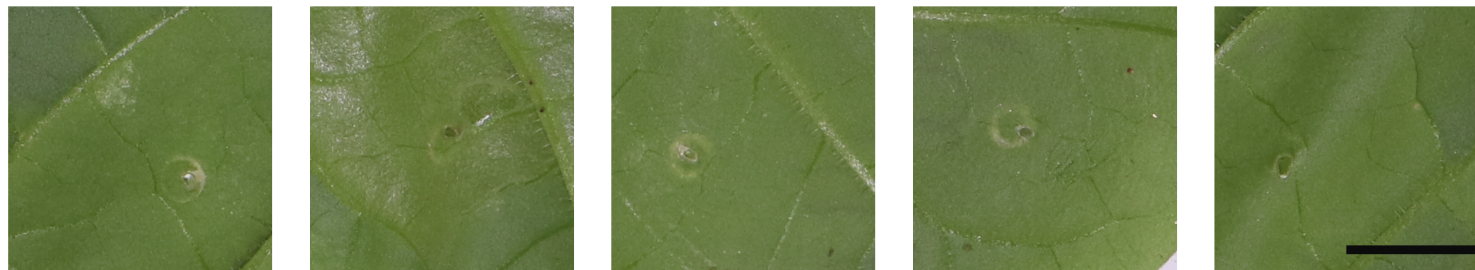*GFP**PuKAN4**PuMYB114**PuKAN4-VP64**PuMYB114+*  
*PuKAN4-VP64*

Supplement: Web_Material_uhaf071 [file web_material_uhaf071.zip › figure s7.pdf]

A

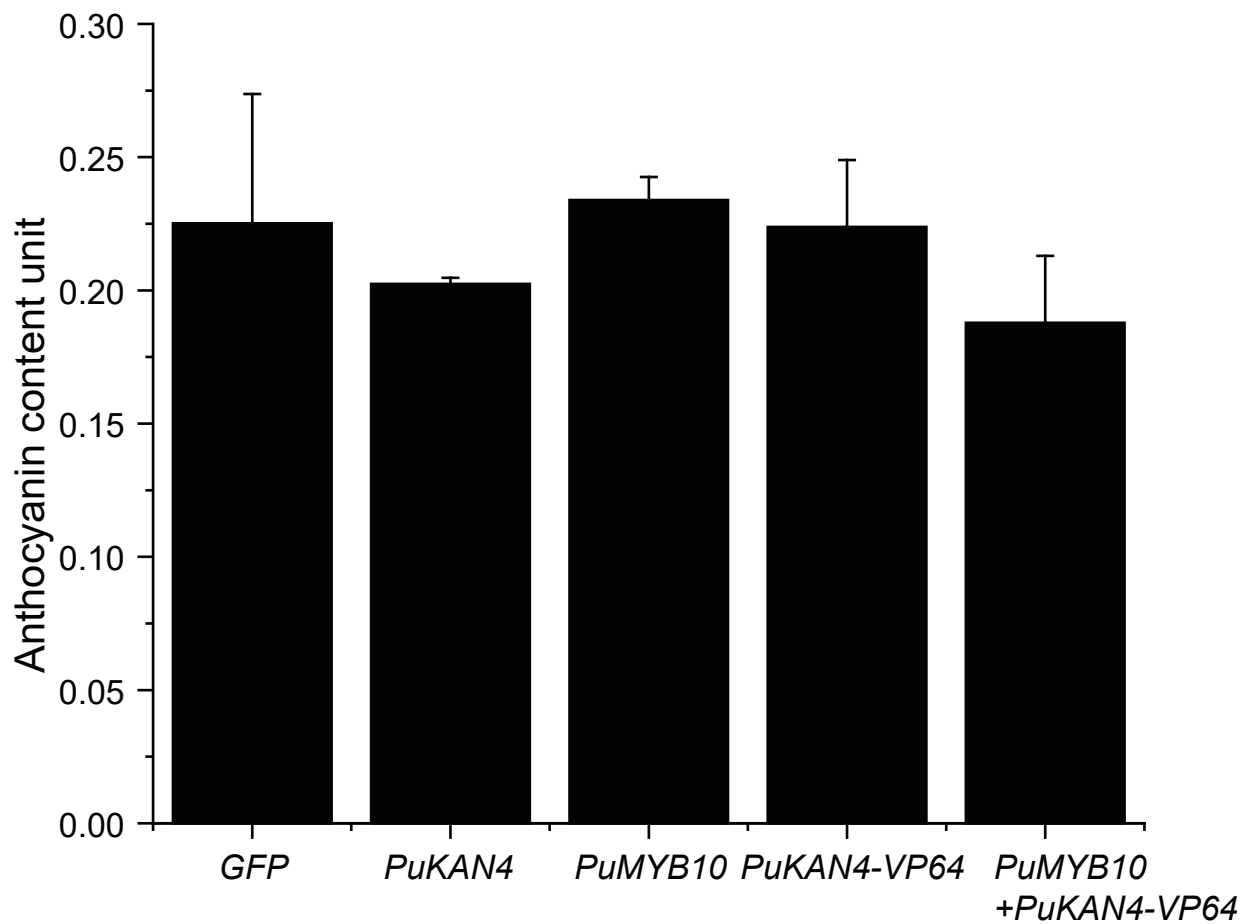

B

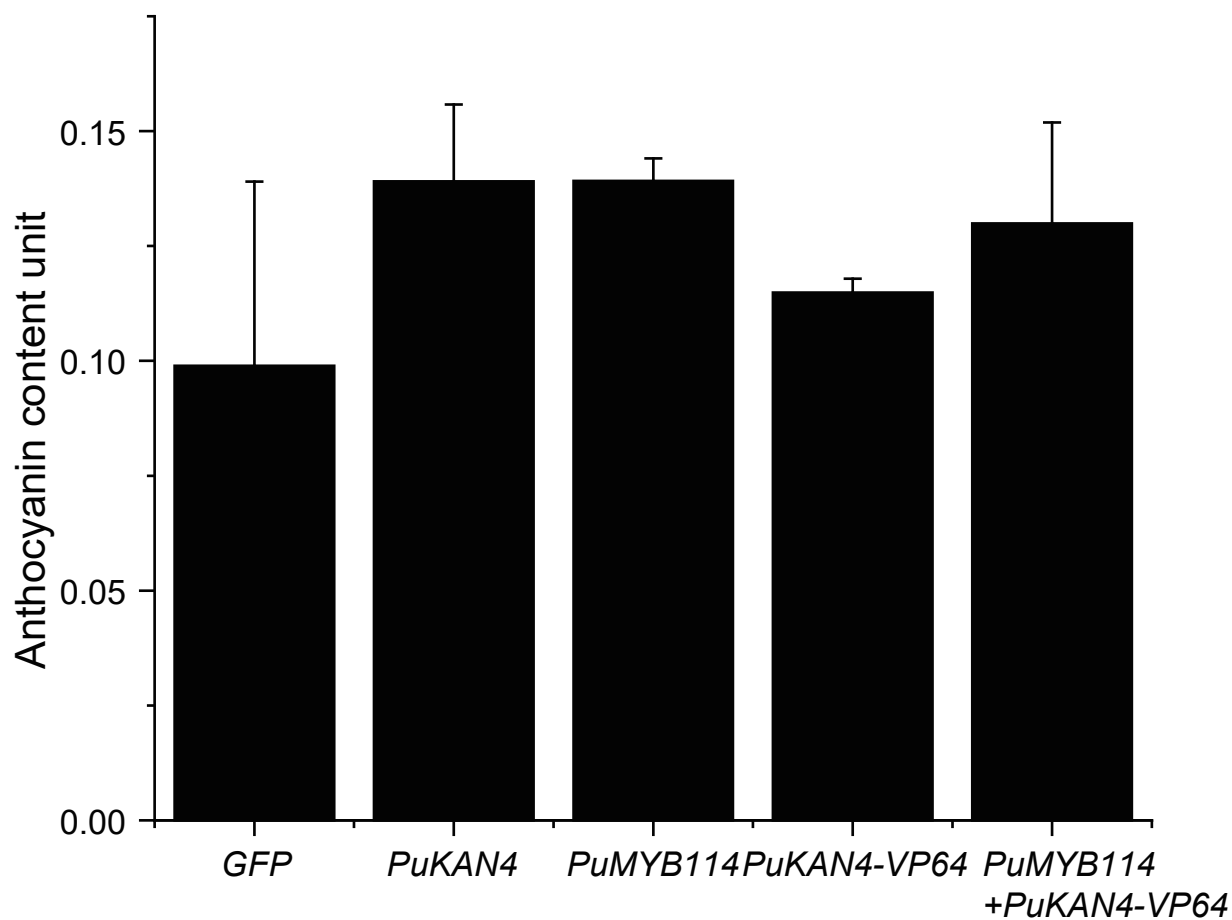

Supplement: Web_Material_uhaf071 [file web_material_uhaf071.zip › figure s8.pdf]

A

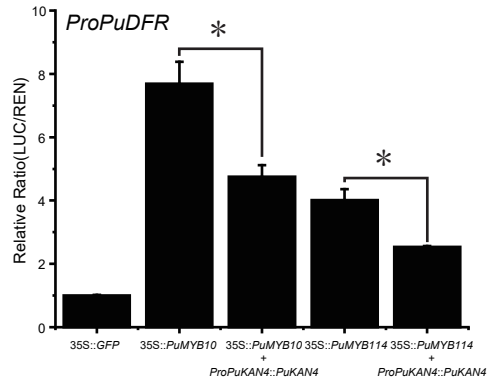

B

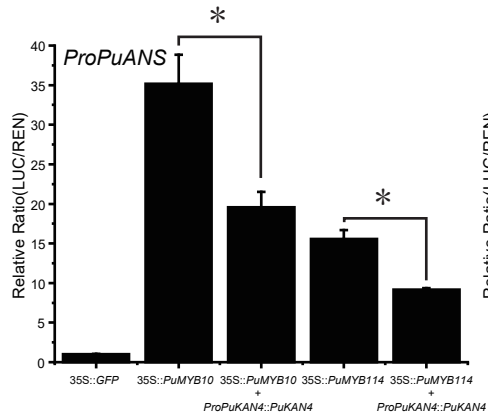

C

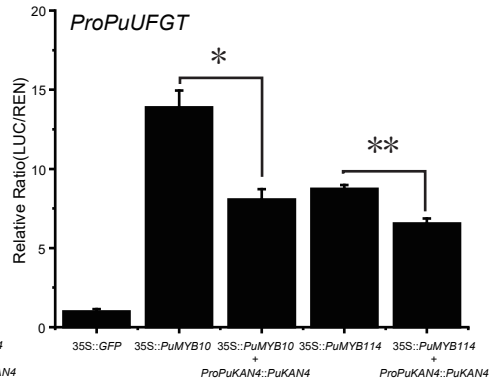

Supplement: Web_Material_uhaf071 [file web_material_uhaf071.zip › figure s9.pdf]
