# Supplementary material for: Characterization of a KANADI-like transcription factor that suppresses pear anthocyanin biosynthesis
Source: Hortic Res. 2025 Mar 3;12(6):uhaf071. doi: 10.1093/hr/uhaf071 (PMC12038239; doi:10.1093/hr/uhaf071)
Supplement: Web_Material_uhaf071 [file figure s2.pdf › figure s2.pdf]

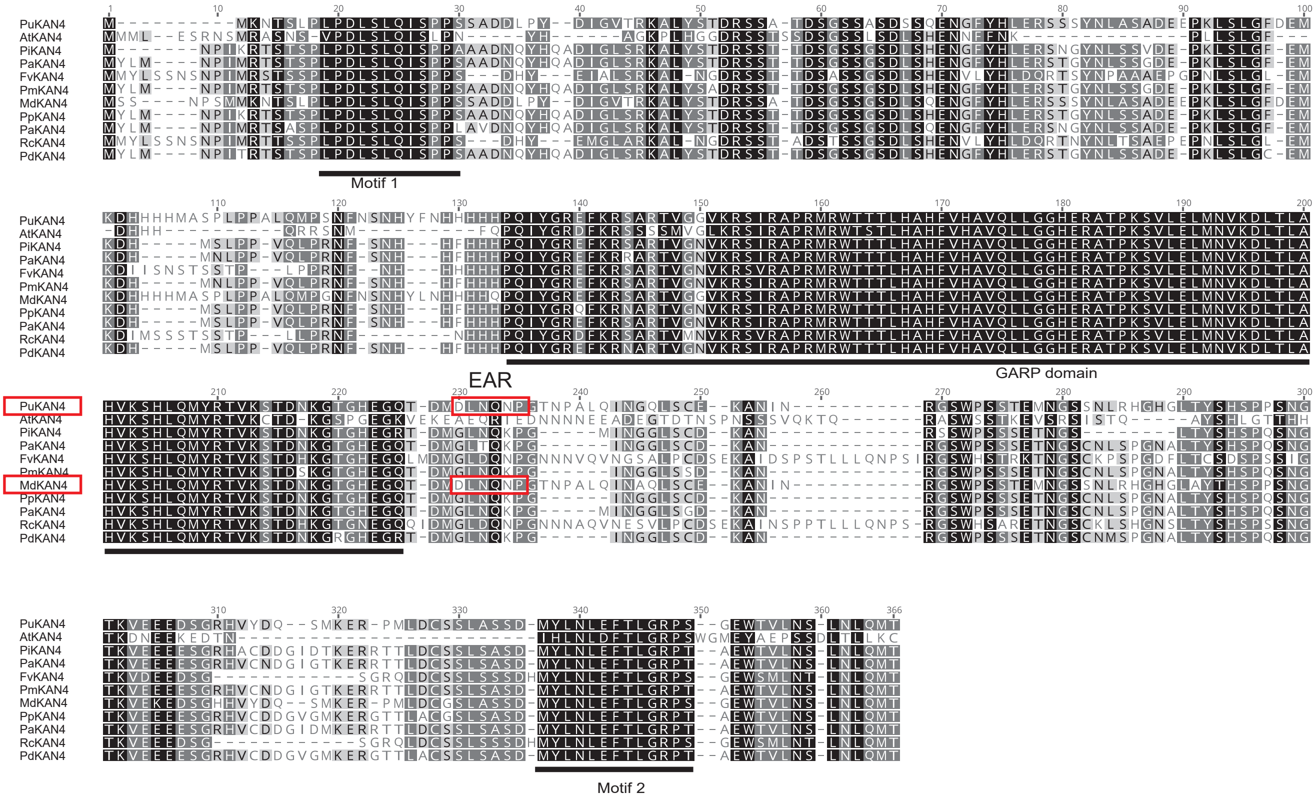
PuKAN4
AtKAN4
PiKAN4
PaKAN4
FvKAN4
PmKAN4
MdKAN4
PpKAN4
PaKAN4
RcKAN4
PdKAN4
Motif 1
EAR
GARP domain
Motif 2
